# “That Line Just Kept Moving”: Motivations and Experiences of People Who Use Methamphetamine

**DOI:** 10.5811/westjem.2022.12.58396

**Published:** 2023-02-25

**Authors:** Callan Elswick Fockele, Sophie C. Morse, Jenna van Draanen, Sarah Leyde, Caleb Banta-Green, Ly Ngoc Huynh, Alina Zatzick, Lauren K. Whiteside

**Affiliations:** * University of Washington School of Medicine, Department of Emergency Medicine, Seattle, Washington; † University of Washington School of Public Health, Department of Health Systems and Population Health, Seattle, Washington; ‡ University of Washington, Department of Child, Family, and Population Health Nursing, Seattle, Washington; § Harborview Medical Center, University of Washington, Department of Medicine, Seattle, Washington; || School of Public Health, University of Washington, Department of Health Services and Population Health, Seattle, Washington; # University of Washington School of Medicine, Addictions, Drug & Alcohol Institute, Department of Psychiatry and Behavioral Sciences, Seattle, Washington

## Abstract

**Introduction:**

Methamphetamine use is on the rise with increasing emergency department (ED) visits, behavioral health crises, and deaths associated with use and overdose. Emergency clinicians describe methamphetamine use as a significant problem with high resource utilization and violence against staff, but little is known about the patient’s perspective. In this study our objective was to identify the motivations for initiation and continued methamphetamine use among people who use methamphetamine and their experiences in the ED to guide future ED-based approaches.

**Methods:**

This was a qualitative study of adults residing in the state of Washington in 2020, who used methamphetamine in the prior 30 days, met criteria for moderate- to high-risk use, reported recently receiving care in the ED, and had phone access. Twenty individuals were recruited to complete a brief survey and semi-structured interview, which was recorded and transcribed prior to being coded. Modified grounded theory guided the analysis, and the interview guide and codebook were iteratively refined. Three investigators coded the interviews until consensus was reached. Data was collected until thematic saturation.

**Results:**

Participants described a shifting line that separates the positive attributes from the negative consequences of using methamphetamine. Many initially used methamphetamine to enhance social interactions, combat boredom, and escape difficult circumstances by numbing the senses. However, continued use regularly led to isolation, ED visits for the medical and psychological sequelae of methamphetamine use, and engagement in increasingly risky behaviors. Because of their overwhelmingly frustrating experiences in the past, interviewees anticipated difficult interactions with healthcare clinicians, leading to combativeness in the ED, avoidance of the ED at all costs, and downstream medical complications. Participants desired a non-judgmental conversation and linkage to outpatient social resources and addiction treatment.

**Conclusion:**

Methamphetamine use can lead patients to seek care in the ED, where they often feel stigmatized and are provided little assistance. Emergency clinicians should acknowledge addiction as a chronic condition, address acute medical and psychiatric symptoms adequately, and provide positive connections to addiction and medical resources. Future work should incorporate the perspectives of people who use methamphetamine into ED-based programs and interventions.

## INTRODUCTION

Methamphetamine use is on the rise nationwide^1^ with an increasing number of emergency department (ED) visits,^2^,^3^ behavioral health crises,^4^–^7^ and deaths associated with use and overdose.^8^ Racial inequities related to methamphetamine use are also increasing, with the highest prevalence of methamphetamine use^8^ and the greatest increases in overdose deaths among American Indians/Alaska Natives. Non-injection methamphetamine use increased 10-fold among Blacks, a much steeper increase than that among White or Hispanic populations.^9^

Methamphetamine is a leading cause of substance-related ED visits.^10^,^11^ The reasons for seeking ED care when using methamphetamine varies with patients requiring anything from medical evaluation for chest pain to sedation and psychiatric evaluation for agitation and psychosis.^12^ In some areas, behavioral crises related to methamphetamine use account for half of psychiatric emergency services visits.^13^ Additionally, patients who inject drugs, such as methamphetamine, seek ED care for injection-related medical complications.^2^ Emergency department visits related to methamphetamine are also likely to involve trauma and/or interactions with law enforcement officers.^14^,^15^ Along with the increase in methamphetamine-related ED visits for medical and psychiatric reasons, emergency clinicians describe methamphetamine use as a significant problem with high resource utilization and risk of violence against staff.^16^,^17^

There is limited literature examining the perspectives of people who use methamphetamine on their health, limiting opportunities to provide care based on patients’ experiences. Among people who use methamphetamine at syringe-access programs across the state of Washington, many were interested in reducing or stopping their use^18^ and wanted assistance addressing their medical and social needs through counseling, treatment, and care navigation.^19^ However, there are no known studies exploring the ED experience of people who use methamphetamine.

Given the increasing prevalence of methamphetamine use and the increasing number of ED visits related to methamphetamine use disorder, it is imperative that EDs consider the best way to serve this population. For patients with opioid use disorder (OUD), EDs have expanded lifesaving buprenorphine prescribing and take-home naloxone programs nationwide,^20^,^21^ activities that undoubtedly have improved the care for patients with OUD.^22^–^24^ In contrast, there is currently a paucity of pharmacotherapy, psychosocial interventions, and harm reduction strategies targeting patients with methamphetamine use disorder. In this study our primary objective was to identify the motivations of people who use methamphetamine and their experiences in the ED. Secondary objectives were to inform key stakeholders, address stigmatizing behavior in healthcare settings, and guide future ED-based approaches.

**Population Health Research Capsule**

What do we already know about this issue?

*Methamphetamine use is rising with more emergency department visits, behavioral health crises, and deaths associated with use and overdose*.

What was the research question?

*What are the motivations of people who use methamphetamine and their experiences in the ED?*

What was the major finding of the study?

*Fifty percent of participants reported that their ‘main drug’ was methamphetamine while 15% preferred methamphetamine and heroin, suggesting that polysubstance use is common*.

How does this improve population health?

*Emergency physicians should recognize the complex motivations for methamphetamine use and provide tools to promote patient wellbeing through trauma-informed care*.

## METHODS

### Study Design and Setting

From April–September 2020, we administered close-ended questionnaires and conducted semi-structured interviews with adults residing in the state of Washington who were at moderate to high risk for methamphetamine use disorder, had presented to an ED within the prior three months, and had access to a phone. The study was approved by the University of Washington Institutional Review Board, and a Certificate of Confidentiality was obtained from the National Institutes of Health.

### Selection of Participants

Participants were recruited through convenience and snowball sampling. Flyers were sent to community substance use treatment clinics, peer support groups within Seattle, WA, supportive housing facilities, office-based opioid treatment programs, opioid treatment programs, and syringe-access program locations. Interested people called our study phone and were screened for eligibility by a trained research assistant (RA). Inclusion criteria included residence in the state of Washington, access to a phone, self-reported ED visit in the prior three months, methamphetamine use in the prior 30 days, and National Institute of Drug Abuse (NIDA)-modified Alcohol, Smoking and Substance Involvement Screening Test (ASSIST) score consistent with moderate or high risk for methamphetamine use disorder.^25^

Those eligible and interested in completing the study next provided verbal informed consent and completed a baseline survey by phone. The study RA directly entered the participant answers into a database using REDCap^26^ electronic data capture tools hosted at the University of Washington. All participants who completed the survey received a $5 gift card. Participants were then invited to be interviewed. We obtained survey data from 25 participants and completed semi-structured interviews with 20 of these participants. The 20 individuals who completed the semi-structured interview provided verbal consent, completed audio recorded interviews over the phone, and received $25 gift cards. After completing an initial set of 10 interviews, we performed purposive sampling of participants who were eligible and completed the baseline survey based upon gender and race for the remaining interview participants to include more diverse perspectives.

### Measurements

During the survey, participants were asked how often they had used methamphetamine in the prior 30 days before undergoing the NIDA-modified ASSIST^25^ to determine risk for methamphetamine use disorder. Participants were next asked to identify their “main drug” to identify their drug of choice. Participants were also asked single-items questions on lifetime intentional fentanyl use and lifetime intentional GHB use. Validated single-item questions about tobacco, vaping, and alcohol were asked. We used the Patient Health Questionnaire-2^27^ and the Generalized Anxiety Disorder-2^28^ to screen for depression and generalized anxiety disorder in the prior two weeks, respectively. The human immunodeficiency virus (HIV) Risk Behavior Survey was used to determine behaviors related to injection, as well as current HIV and hepatitis C virus status. Demographic information, including age, gender, employment, and housing status, were collected. Qualitative semi-structured interviews focused on methamphetamine use, ranging from the causes behind their initial use to current use patterns, as well as on ED experiences, focusing on the patient’s last ED visit related to methamphetamine use, their experiences seeking and accessing care, and their thoughts regarding how the ED could meet their needs. The interview guide was refined iteratively, and the final guide is included as an appendix.

### Analysis

Using descriptive statistics, we analyzed the survey results for participants who completed the survey and the semi-structured interview. The quantitative analysis was restricted to the 20 participants who completed both the survey and the interview. Semi-structured interviews using a standardized interview guide were recorded, transcribed, deidentified, and uploaded to the qualitative data management software Dedoose (SocioCultural Research Consultants, LLC, Manhattan Beach, CA). We used a modified grounded theory framework^29^,^30^ to continuously collect and analyze the qualitative data. The grounded theory framework^29^,^30^ allows the results to emerge from the data without a preconceived hypothesis. Therefore, coding of the manuscripts proceeded in an iterative fashion allowing data and codes from the initial manuscripts to inform the results codebook.

Specifically, we conducted three initial interviews with an interviewer (LH) who had experience conducting semi-structured interviews and working with the target population. After these initial interviews, three members of the research staff (LH, SM, AZ) each independently reviewed two transcripts and inductively developed and applied codes to the transcript.^31^ This process iteratively refined the codebook. These members and the principal investigator (LW) then met as a group until consensus was achieved on the codebook, with LW as the arbitrator. Finally, subsequent semi-structured interviews were conducted by the same trained interviewer (LH) until thematic saturation was reached.

## RESULTS

### Quantitative Results

Of the 25 participants who completed the survey, we interviewed 20 adults who met inclusion criteria (Tables 1 and 2). The mean age of our participants was 41.5 years (SD 8.7 years), and most participants were White cisgender men. All participants reported experiencing homelessness at some point in their lifetime while 40% were unstably housed at the time of the interview. Ninety percent were unemployed. Many participants reported current polysubstance use. Among this sample of 20 people who reported currently using methamphetamine, 10 (50%) reported that methamphetamine was their drug of choice, while 45% reported methamphetamine combined with something else to be their preferred drug. Sixty-five percent had injected methamphetamine in the prior month, and 55% reported that their main route of administration was smoking. Thirty percent had visited the ED because of methamphetamine use in the prior 30 days. Most respondents noted physical and/or psychiatric symptoms associated with methamphetamine overdose, or “overamping,” in the prior 12 months.

### Qualitative Results

Our study’s major theoretical contribution is that participants described a shifting line that separates the positive attributes from the negative consequences of using methamphetamine. This was best summarized by one individual, who explained: “I kept drawing lines of delineation. . . .It was just going to be when I was hooking up, and then it was just going to be on weekends. Then, it was just going to be not on workdays. And then it was going to be I was never going to inject. That line just kept moving.” This line also represents interviewees’ complex, occasionally paradoxical, and often shifting experiences with methamphetamine, including enhancing function while also inducing crippling paranoia, fostering friendship while also leading to unequal relationships, and addressing untreated trauma while also exacerbating it. Several themes straddled this line: 1) hypervigilance and overamping; (2) socialization and isolation; (3) treatment and withdrawal; and (4) experiences in the ED.

### Hypervigilance and Overamping

Many interviewees reported initially using methamphetamine to enhance their function, whether it was cleaning, working, or studying, and to provide protection in harsh conditions like homelessness. However, this hypervigilance often led to “overamping” when a participant might have felt that they were overdosing, “paranoid,” and “exhausted” (Table 3).

### Socialization and Isolation

Participants described how methamphetamine originally improved their social interactions. They frequently started using with friends in social settings or to enhance sex. However, continued use regularly led to isolation and “stopping participation in life.” Individuals experiencing methamphetamine-induced paranoia felt uncomfortable around others, and repeated bingeing (ie, multiple days of consecutive use) often contributed to losing family, friends, jobs, property, and “personality.” Others recounted how individuals capitalized on their drug use, preyed on their vulnerabilities, and fostered unequal relationships (Table 4).

### Treatment and Withdrawal

Many interviewees used methamphetamine to self-medicate, stabilizing their mental health, numbing their senses to escape difficult circumstances, and counteracting the negative effects of other drugs. However, the increasing need to use methamphetamine to combat withdrawal symptoms led participants to “hustle” and engage in increasingly risky behaviors, like sex work, to obtain the resources to purchase enough to avoid feeling sick (Table 5).

### Experiences in the Emergency Department

Interviewees often experience stigmatizing healthcare interactions because of their methamphetamine use. Many described undertreatment of pain, difficulty obtaining intravenous access, unhelpful referrals, and traumatizing experiences, particularly while intoxicated with methamphetamine. Because of these overwhelmingly frustrating experiences, participants anticipated difficult interactions with healthcare clinicians, frequently leading to combativeness, avoidance of the ED, and downstream medical complications (Table 6). Nevertheless, methamphetamine use often drives patients to EDs, where they would like to receive resources, shelter, and treatment (Table 7).

## DISCUSSION

Experiences with overamping, isolation, and withdrawal mirror the current literature describing the negative consequences of use,^32^ but participants also explored how methamphetamine can enhance function and strengthen relationships. This “moving” line between methamphetamine’s risks and benefits highlights the need for nuanced conversations about substance use in medical settings. People who use methamphetamine often want to reduce their use, but their motivation and goals are fluid.^19^ Emergency physicians should recognize the complexity of patients’ motivations and provide tools to promote wellbeing. They should aspire to provide trauma-informed care^33^ to those who use drugs by better understanding each patient’s unique history and recognizing the health effects of stigma.^34^

Participants frequently acknowledged the dangers of methamphetamine and wanted help but purposefully avoided medical care because of the perceived discrimination from healthcare staff. Many cited disrespectful interactions, undertreatment of pain, difficulty obtaining intravenous access, unhelpful referrals, and traumatic experiences in the ED related to their methamphetamine use. Interviewees hoped for, but rarely encountered, clinicians who acknowledged addiction as a chronic condition, addressed symptoms adequately, and provided positive connections to outpatient resources. This stigma experienced by people who use methamphetamine mirrors stigma experienced by people who use opioids.^35^ Moreover, many methamphetamine-related ED visits for behavioral health concerns include chemical and/or physical restraints, which can feel dehumanizing to patients.

Emergency physicians can learn from community harm reductionists at syringe service programs and safe consumption sites about how to change this culture and create a protected space for people who use methamphetamine.^36^ The distribution of safer use supplies, such as syringes and pipes, decreases risky behaviors and the spread of infectious diseases while promoting more collaborative medical interactions.^37^–^45^ Because methamphetamine use is associated with high-risk sexual practices, clinicians can also consider sexually transmitted infection testing, treatment, and prevention services. Whether or not these services could be expanded to emergency care settings should be further explored.

Although not widespread, harm reduction principles have been successfully integrated as pilot programs into traditional clinical settings, which could be used as models in other environments. One hospital system created a multidisciplinary and interprofessional care conference to expand treatment options for patients with substance use disorders needing prolonged antibiotic treatment for conditions like endocarditis and osteomyelitis.^46^ As part of their efforts to improve access to addiction care in emergency departments, CA Bridge, a program of the Public Health Institute in Oakland, California, has created adaptable materials on harm reduction kits, discharge instructions, strategies for hospital settings, and order sets based upon the experiences of selected clinical partners.^47^–^50^

Lastly, as in other published work,^51^ participants expressed interest in accessing treatment and reducing their methamphetamine use. Although an effective pharmacotherapy for methamphetamine use has not yet been developed, there are several effective, yet underutilized, psychosocial treatments for methamphetamine use disorder. Contingency management^52^ reinforces positive behavioral change with rewards. Examples of incentivized behaviors include abstinence, engagement in therapy sessions,^53^ and harm reduction.^54^ Rewards typically include prize draws in cash or gift cards of escalating value. Although contingency management can be effective on its own, it can also be paired with the community reinforcement approach,^55^ which uses social, recreational, familial, and vocational reinforcers to help patients engage in non-substance-use related activities and communities, so they can find meaning in a lifestyle that does not revolve around substance use.^56^ A recent meta-analysis showed that contingency management coupled with the community reinforcement approach was the only evaluated treatment associated with decreased substance use at the longest follow-up time and increased engagement in treatment for individuals with stimulant use disorder.^57^ Contingency management has been successfully implemented in homeless shelters,^58^ community centers,^54^ primary and specialized care clinics,^59^,^60^ and sober living arrangements.^61^ Emergency physicians should consider creating referral pathways for patients who use methamphetamine in partnership with agencies providing these evidence-based interventions.

## LIMITATIONS

The objective of this study was to identify the motivations of people who use methamphetamine and their experiences in the ED to guide future ED-based approaches. However, the results may only be applicable to the geographic location of the study population, which only included residents of the state of Washington. We used a convenience sampling frame to recruit participants, which may have introduced bias. Specifically, recruitment and interviews did not take place in person; therefore, this study may not have captured the voices of those with high social needs without access to a phone. Additionally, questionnaire data, including recent ED visits and substance use history, were self-reported and could not be confirmed with the patient’s electronic health record or through drug testing. Lastly, the study was conducted at the beginning of the coronavirus disease 2019 pandemic, while the “stay home, stay healthy” order was in place,^62^ which may have influenced participants’ perceptions of their medical care.

## CONCLUSION

Methamphetamine use drives patients to EDs, where they often feel stigmatized and are provided little assistance. Emergency physicians can use trauma-informed care to change this culture and create a healing space for people who use methamphetamine. They can offer ultrasound-assisted peripheral line placement and treat symptoms of overdose, withdrawal, and pain. Using harm reduction principles, EDs can provide HIV and hepatitis C testing and distribute safer use supplies. Physicians can partner with a multidisciplinary team to improve access to social services and transitions of care to addiction treatment in the community. Future work should incorporate the perspectives of people who use drugs into ED-based programs and interventions.

## Supplementary Information



## Figures and Tables

**Table 1:** Demographics, substance use characteristics, and medical characteristics of interviewees.

	N=20 (%)
Demographics	
Age (mean)	41.5+/−8.7
Female	6 (30)
Male	11 (55)
Other gender	3 (15)
Race/ethnicity	
White	12 (60)
Black	6 (30)
Hispanic/Latinx	4 (20)
Two or more races	3 (15)
Prefers not to answer	1 (5)
Currently experiencing homelessness	8 (40)
Unemployed	13 (65)
Substance use characteristics	
Non-methamphetamine substance use in the prior 30 days	
Cigarettes or e-cigarettes	15 (75)
Alcohol	10 (50)
Heroin	9(45)

Substance Use Characteristics	

Non-methamphetamine substance use in the prior 30 days	
Lifetime intentional use of fentanyl	3 (15)
Lifetime intentional use of GHB	10 (50)
Injected any drug more than once per day in the prior month	8 (40)
Lifetime opioid overdose	6 (30)
Depression in last two weeks (PHQ-2 >=3)	15 (75)
Anxiety in past two weeks (GAD>=3)	18 (90)
HIV + (sample size is n=19)	3 (16)
HCV +	4 (20)

*PHQ-2*, Patient Health Questionnaire-2; *GAD*, General Anxiety Disorder scale; *HIV*, human immunodeficiency virus; *HCV*, hepatitis C virus. *GHB*, gamma hydroxy butyrate

**Table 2:** Me thamphetamine use characteristics of interviewees.

	N=20 (%)
Methamphetamine use in the past 30 days	20 (100)
Injected methamphetamine in the last 30 days	13 (65)
Self-reported “main drug”	
Methamphetamine “by itself”	10 (50)
Methamphetamine combined with:	8 (40)
Heroin	3 (15)
Alcohol	1 (5)
Cannabis	2 (10)
Cocaine	1 (5)
GHB	1 (5)
Other main drug	2 (10)
High risk for methamphetamine use disorder (NM-ASSIST >= 27)	19 (95)
Preferred method of using methamphetamine	
Smoking	11 (55)
Injecting	9 (45)
Experiences using methamphetamine	
In the last 12 months, have you ever felt like you were having a heart attack, stroke, or seizure while on meth? (yes)	9 (45)
In the last 12 months, have you ever had a time when you felt like you were losing your mind, manic, or psychotic while on meth? (yes)	14 (70)
In the last 12 months, have you been to an emergency room because of medical or psychiatric problems related to meth? (yes)	13 (65)

NM-ASSIST, National Institute on Drug Abuse modified Alcohol, Smoking and Substance. Involvement Screening Test. *GHB*, gamma hydroxy butyrate

**Table 3:** Interviewee experiences that describe hypervigilance and overdosing (overamping).

Hypervigilance	
Enhanced functioning	“It was all really to get through college, and I got my degree. It helped me stay up to study for exams.” (#40) “With the meth I’m functional. [Without it] I might miss being able to make a list of five things to do and actually accomplishing four or five of them.” (#46)
Provide protection	“Being hypervigilant also puts me in a place where I don’t put myself into situations that I can be jailed or fucked up by cops.” (#25) “I wanted to be aware and coherent of what was going on around me. I didn’t like the nodding and falling just anywhere.” (#40)
Overamping	
Paranoia	“Lots of paranoia is involved and just confusion, like I get caught in a loop and I can’t stop doing, digging for something, trying to fix something. I just get stuck on a path that I can’t stop doing.” (#7)
Exhausted	“We don’t recognize where we’re at and recognize where our limits are. We don’t sleep, we don’t eat for days. We don’t really recognize that our bodies haven’t rested.” (#4)

**Table 4:** Interviewee experiences that describe socialization and isolation.

Socialization	
Friendship	“There was a long period of time it was actually fairly fun. . . . There were lots of social circles that we’d use and have fun, but that quickly faded.” (#7) “The social aspect of it got me doing it again. And shooting is just a fun way to do it compared with smoking for me, so other people got me back into it.” (#29)
Sexual augmentation	“Sex would be the trigger for the longest time. . . . It was like a whole different animal, the intensity, the rush, the sexual feelings related to it are totally different.” (#7) “When you’re with someone that’s not on it and you are really, really on it, you just don’t have like the same goals in mind or just the same urgency to get done what you want to get done.” (#33)
Isolation	
Uncomfortable around others	“Meth is a drug that causes you to socially isolate and social distance. People are paranoid.” (#4)
Loss	“I only participate in getting high. I’ve got a whole bunch other things I could participate with. I got kids and grandkids and family. . . . I don’t want to do nothing but get high.” (#7) “I lost all my friends, all my surroundings around me, all my coworkers. I lost communication with relatives and people that I had in my life. . . . I don’t know why we even continue criminalizing [drugs] because I’m already a prisoner.” (#41)
Unequal relationships	“Living on the road, being homeless off and on, and now it’s like total dependency, so there are places I’ll get housed at because like a guy or an older guy would help me out for a little bit . . . but then they’re very manipulative.” (#26)

**Table 5:** Interviewee experiences that describe treatment and withdrawal.

Treatment	
Mental health	“It maybe relates to a specific disorder . . . maybe like ADD or ADHD . . . I want to say that using meth . . . putting the hyperactive mind with the hyperactive drug to stimulate kind of almost reduces . . . that hyperactivity.” (#19) “It’s more than just for fun because it stabilizes my mood disorder.” (#40)
Escape	“I had lost my job, my partner. . . . We were in a kind of a low and violent point, and it was an escape. . . . I really think the whole reason I started was self-medicating.” (#15) “Definitely coping and also helps me drown out . . . Memories or emotions. . . . It’s a ritual routine now.” (#26)
Negative effects of other drugs	“You get the meth rush over the black. . . It goes back and forth, like you’ll feel the numbing effect from heroin, the slow effect, and then it’ll switch over to the meth high, the racy, euphoric kind of feeling that you get from meth.” (#29)
Withdrawal	
Symptoms	“Now, unfortunately, when I do stop, it makes me horribly sick. . . I don’t really have the luxury of just choosing not to do it anymore.” (#12)
Hustling	“A typical day, like I wake up, I do a shot of heroin, smoke some meth, go hustle, smoke some more meth, do another shot, go hustle, and do the same thing, then go to sleep.” (#10) “Usually, I’ll panhandle most days and get enough money to maintain not being sick throughout the day. . . . My day revolves around having the shots to do.” (#29) “I have kind of a boyfriend, and he does leave meth for me when he leaves.” (#46)

**Table 6:** Interviewees’ negative experiences in the emergency department.

Stigmatizing care	“As soon as they find out that, yes, it was 100 percent drug-related, I get treated differently.” (#29) “Maybe after some work with this population, maybe people give some sort of a numbness . . . like they don’t see you are regular [person] or they see [you as], ‘She’s already overdosed and so why should we care about you?’” (#41)
Undertreatment of pain	“We’ll go through these procedures with absolutely no pain med at all. . . . And they feel like I’m asking to be sent home with pain meds, [thinking] I’d obviously abuse them. So I never ask to bring any home.” (#29)
Difficulty obtain IV access	“I’m terrified of needles when someone else is doing it, and, then, with not having very many veins to poke . . . They have to get an ultrasound, so it’s a really big ordeal when I go [to] a hospital and have to have blood taken from me.” (#12)
Unhelpful referrals	“The doctor said I need to follow up with this [a community help line]. [But I’m thinking,] ‘How can I follow up with this if you’re not giving me no more information that I already had before I came in here?’” (#46)
Traumatizing experiences	“When I was walking to the emergency room, fire trucks and shit like that . . . fucking irritate my goddamn brain cells. I come out and certain sound effects and shit like that, paranoia. (#34) “I don’t know how many times I’ve gone to the hospital, scared out of my mind, and I was high, and they treated me unfairly because I was high.” (#39)
Combativeness	“And then they find out that I’m an addict, and it all goes downhill. . . . Maybe I get like a little bit of like a bad attitude. . . If I know that this person’s going to be mean to me because everybody else has been, then I’m going to be mean initially anyway.” (#10)

*IV*, intravenous.

**Table 7:** Interviewees’ positive experiences in the emergency department.

Resources	“Give them some resources, whether or not they said yes or no.” (#26) “About places to get into rehab, places for wound care, like a place to heal up afterwards if you’re homeless. Like maybe the needle exchange. Just like information of things that addicts and homeless people could really use.” (#43)
Shelter	“When I have done treatment, it was when I was homeless, so after the treatment [I’d] get released right back to the same situation. No place to go, no home. You can refer me to all these outpatient places and tell me I need X amount of meetings, but once I go to my classes and go to my meeting, now where do I go?” (#46)
Treatment	“I think ERs are probably overwhelmed, and they don’t need a bunch of people coming in saying, ‘Where can I go to rehab?’ But if they don’t have anywhere else to go . . .” (#15) “The one thing that I’ve found that helped me when I was trying to quit was my doctor prescribed me methylphenidate . . . And I don’t understand why that’s not utilized more often because for opiates they use like Suboxone and methadone.” (#20)
