# Supplementary material for: “That Line Just Kept Moving”: Motivations and Experiences of People Who Use Methamphetamine
Source: West J Emerg Med. 2023 Feb 25;24(2):218–27. doi: 10.5811/westjem.2022.12.58396 (PMC10047723; doi:10.5811/westjem.2022.12.58396)
Supplement: Supplementary file 1 [file wjem-24-218-s001.docx]

*Interview Guide*

Introduction

Thank you for volunteering for this interview today. My name is <NAME> and I am the <research role> on this project. Our conversation will take around 30 minutes of your time, more or less. At the end of your conversation we will send you a $30 electronic gift card as a thank you.

I am working with a team of healthcare providers and researchers at the University of Washington with a goal of trying to improve care for people who use meth. To get a better understanding of this, I’m going to talk with you today about your experiences around substance use, health care, and COVID-19. This talk is meant to be non-judgmental and open ended. We see you as an expert of your own health and want to understand your honest story and unique experiences. Some of the topics we touch on might be difficult to talk about and we appreciate your willingness to share as much of your story as you feel comfortable with. Part of today’s talk will reference the survey you completed and I might ask you to expand on some of those answers. Our conversation today is confidential and will not be linked to you or your health care records and cannot get you in legal or medical trouble. We will be using a tape recorder to record this conversation, however after the interview we will keep this transcript in a password protected datafile and remove any links to your identity. We will keep the recording of your name and consent separate from the actual interview. We respect your privacy and if you want to pause or skip a particular topic or question just let us know. Are you ready to start?

Great, please state your name and that you consent to this audio recording.

This interview will mainly focus on your experience with meth but we understand this might not be your main drug. In the survey you mentioned your ‘main drug’ is ______________________. Tell us a little about that? When did you last use? How often you do use? What does a day that you are using look like? What do you like about using ________? What do you not like about using __________?

Motivations:

1. Tell us about how/when you started using methamphetamine?
2. What were the social circumstances that you started using and has this changed? How would you describe the way you use now? Probe: Do you usually use by yourself or with others?
3. Your main drug is _______________ . Tell me about why you use ________________?
4. Tell us about what a typical day looks like when you are using. How much do you use? How often? Why do you smoke instead of inject? (or vice versa)
5. Tell us about drug use in your family?
6. It sounds like you first began to use to _______, but now it seems that your use of meth is causing you some grief. If this is the case, and correct me if I am wrong, why do you continue to use?

Experiences:

1. Can you tell us about the last time you used meth. What was the benefit of using methamphetamine at that time? Did you combine with another drug like heroin? If yes, describe that for us. Probe some people use opioids to “come down” from methamphetamine. What are your thoughts on this? Do you do this?
2. Tell us about the last time you had a methamphetamine overdose? Probe in the survey you mentioned that you [insert response to meth overdose questions here]. Tell us about that experience. Did you go to the ER?
   1. Tell us about the last time you felt like you were losing your mind, manic, or psychotic while on meth?
   2. Tell us about the last time you were having a heart attack, stroke, or seizure while on meth?
   3. In the last 12 months, have you been to an emergency room because of medical or psychiatric problems related to meth?
   4. What about your use was different this time? What do you think caused you to become overamped?
   5. How do you respond when you are overamped? What do you do to bring yourself down?
3. In the survey you mentioned exchanging sex for drugs, money, or other resources in your lifetime.
   1. Tell us about your experience engaging in sex work?
   2. How has meth use influenced your engagement in sex work?
4. You mentioned in the survey that you have been prescribed ______ in your lifetime. When is the last time you took this medication? What did you use this for?

Care Transitions (Health Care)

1. Tell us about your last memorable ED visit related to methamphetamine use. Probe What was the reason for your visit? Were you experiencing an overdose? Did someone come with you? Did you see a mental health worker or a psychiatrist? Were you restrained or receive medications that made you sedated? Tell us about this experience.
2. Can you tell us about your experiences seeking and accessing care for drug use recently? How do you think the ED can help you with treatment for methamphetamine use? How about for heroin use? Probe: Walk me through what it was like to enter treatment for methamphetamine and opioids in combination.
   1. What does “recovery” mean to you? What does “treatment” mean to you?
   2. What was helpful for you during your inpatient treatment?
3. What would you want your ER care team to know about you to help you access the care that you want?
   1. What would you want out of an ER visit when it comes to your meth use?
   2. What kind of questions, support, or resources would you want offered at the ER?
   3. What tangible thing you like to leave the ER with? (resources, having talked to someone, follow up appointment, etc)
   4. How can the ER meet you where you are?

COVID-19

1. COVID has changed life in a way that no one saw coming. How has COVID impacted you? Have you been worried about contracting COVID and have you been tested or tried to get testing for COVID? Has anyone you known gotten COVID? What do you see as your risk factors for COVID?
2. How has COVID changed the way you use drugs? In the last couple of months, how you used more or less of anything? Where do you get drugs from and has that changed?
